# Same same but different: dopamine transporter SPECT on scanners with CZT vs. NaI detectors

**DOI:** 10.1186/s13550-023-00973-8

**Published:** 2023-03-22

**Authors:** Felix Thiele, Franziska Schau, Julian M. M. Rogasch, Christoph Wetz, Stephanie Bluemel, Winfried Brenner, Holger Amthauer, Catharina Lange, Imke Schatka

**Affiliations:** 1 Department of Nuclear Medicine, Charité – Universitätsmedizin Berlin, corporate member of Freie Universität Berlin and Humboldt-Universität Zu Berlin, Berlin, Germany; 2 Berlin Institute of Health (BIH) at Charité – Universitätsmedizin Berlin, Berlin, Germany

**Keywords:** Dopamine transporter, SPECT, FP-CIT, CZT, Normal database

## Abstract

**Background:**

The aims of this study were to establish a normal database (NDB) for semiquantification of dopamine transporter (DAT) single-photon emission computed tomography (SPECT) with [^123^I]FP-CIT on a cadmium zinc telluride (CZT) camera, test the preexisting NaI-derived NDB for use in CZT scans, and compare the diagnostic findings in subjects imaged with a CZT scanner with either the preexisting NaI-based NDB or our newly defined CZT NDB.

**Methods:**

The sample comprised 73 subjects with clinically uncertain parkinsonian syndrome (PS) who prospectively underwent [^123^I]FP-CIT SPECT on a CZT camera according to standard guidelines with identical acquisition and reconstruction protocols (DaTQUANT). Two experienced readers visually assessed the images and binarized the subjects into “non-neurodegenerative PS” and “neurodegenerative PS”. Twenty-five subjects from the “non-neurodegenerative PS” subgroup were randomly selected to establish a CZT NDB. The remaining 48 subjects were defined as “test group”. DaTQUANT was used to determine the specific binding ratio (SBR). For the test group, SBR values were transformed to z-scores for the putamen utilizing both the CZT NDB and the manufacturer-provided NaI-based NDB (GE NDB). A predefined fixed cut-off of -2 was used for dichotomization of z-scores to classify neurodegenerative and non-neurodegenerative PS. Performance of semiquantification using the two NDB to identify subjects with neurodegenerative PS was assessed in comparison with the visual rating. Furthermore, a randomized head-to-head comparison of both detector systems was performed semiquantitatively in a subset of 32 out of all 73 subjects.

**Results:**

Compared to the visual rating as reference, semiquantification based on the dedicated CZT NDB led to fewer discordant ratings than the GE NDB in CZT scans (3 vs. 8 out of 48 subjects). This can be attributed to the putaminal z-scores being consistently higher with the GE NDB on a CZT camera (median absolute difference of 1.68), suggesting an optimal cut-off of -0.5 for the GE NDB instead of -2.0. Average binding ratios and z-scores were significantly lower in CZT compared to NaI data.

**Conclusions:**

Use of a dedicated, CZT-derived NDB is recommended in [^123^I]FP-CIT SPECT with a CZT camera since it improves agreement between semiquantification and visual assessment.

## Background

Dedicated cardiac gamma camera systems equipped with semiconductor detectors of cadmium zinc telluride (CZT) have been in clinical use for over a decade. Compared to conventional NaI-based systems, these CZT cameras equipped with dedicated collimators offer a higher sensitivity and better energy resolution when performing myocardial perfusion single-photon emission computed tomography (SPECT) [1–7]. With the introduction of cameras equipped with wide-field CZT detectors [8–10], this technology can be applied to a vast variety of nuclear medicine examinations, such as brain SPECT with N-ω-fluoropropyl-2β-carbomethoxy-3β-(4-I-123-iodophenyl)nortropane ([^123^I]FP-CIT).

[^123^I]FP-CIT SPECT is a well-established imaging technique for in-vivo visualization of the pre-synaptic dopaminergic function. Therefore, it is used as a diagnostic tool for examining the nigrostriatal integrity of patients showing symptoms of clinically uncertain parkinsonian syndromes (PS) [11–13]. To complement visual reading of SPECT images and improve differentiation between normal and abnormal findings, the European Association of Nuclear Medicine (EANM) procedure guideline for [^123^I]FP-CIT SPECT proposes semiquantitative analysis of the tracer binding on the acquired SPECT scans [12, 14–18]. By calculating the specific binding ratio (SBR), further information about the [^123^I]FP-CIT binding to the dopamine transporter (DAT) in the striatum and its subregions can be provided. In this context, the contralateral putamen is the most relevant of these subregions for the differentiation between neurodegenerative and non-neurodegenerative PS [19].

SBR analysis requires a normal database (NDB) comprised of healthy controls and based on images acquired with the same system-specific acquisition and reconstruction parameters to serve as a standard of reference. However, the existing normal databases provided by the manufacturers have been established only for NaI-based camera systems so far. Furthermore, it remains unclear, what impact the high-energy photons of ^123^I might have on semiquantification in CZT-based systems optimized for ^99m^Tc.

Therefore, the aims of the present study were to (i) prospectively establish a normal database for semiquantification of brain SPECT imaging with [^123^I]FP-CIT on our CZT camera, (ii) test the preexisting NaI-derived normal database for use in CZT scans, and (iii) compare the diagnostic findings in subjects imaged with a CZT scanner with either the preexisting NaI-based normal database or our newly defined CZT normal database.

## Methods

### Study sample

Seventy-three subjects were prospectively included (46 males, 27 females; median age, 67 years, interquartile range [IQR], 61 to 75 years; range, 25 to 83 years) who underwent [^123^I]FP-CIT SPECT in our center from March 2018 to September 2019 with clinically uncertain neurodegenerative parkinsonian syndrome.

### Image acquisition and reconstruction

Prior to injection of [^123^I]FP-CIT, subjects were administered a single dose of 600 mg sodium perchlorate solution for thyroid blockage. [^123^I]FP-CIT (median, 180 MBq; IQR, 177 to 185 MBq) was injected intravenously. SPECT data acquisition started 3.4 h (IQR, 3.1 to 3.8 h) after tracer injection. This time point was chosen to guarantee that subjects could be included in the crossover subgroup as well, still fitting into the EANM guideline’s recommended time frame [12].

Imaging was performed in all patients using a general purpose two-head SPECT system equipped with CZT detectors and a wide-energy high-resolution (WEHR) collimator (Discovery™ NM/CT 670 CZT, GE Healthcare). Images were acquired with sixty views per detector à 30 s in 3° steps over a 360° orbit, 128 × 128 × 120 matrix and zoom of 1.33. The photo peak was set to 159 keV ± 5%. Overall acquisition time was 30 min. Patient-detector distance was minimized manually. For SPECT image reconstruction, a Xeleris workstation running DaTQUANT v1.0 (GE Healthcare) was used. Scans were reconstructed with a 128 × 128 × 128 Matrix (3.323 mm pixel size of an isotropic voxel) and by using the OSEM algorithm with default parameters (2 iterations and 10 subsets with a Butterworth filter with a cut-off of 0.6 cycles/cm and power 10) and Chang attenuation correction (μ = 0.12/cm); additional scatter correction was not performed. These reconstruction parameters were chosen with regard to the used presets of the manufacturer’s NDB and to ensure high comparability.

For evaluating the SBR and z-score differences, and, thus, diagnostic findings between both NaI and CZT cameras, 32 out of the 73 subjects agreed to undergo additional imaging on the same day with a comparable conventional NaI-based system (Discovery™ NM/CT 670 DR, GE Healthcare). Acquisition and reconstruction parameters were identical for the NaI system. Individuals with imaging on both camera systems were labeled as “crossover group”. Subsequent SPECT imaging with the CZT and NaI system was performed in a randomized order to account for photon decay. By chance, half of the crossover group was first scanned with the CZT system (*n* = 16) and the other half with the NaI system (*n* = 16). When imaging subjects on the CZT camera first, there was a median delay of 10 min (IQR, 7.5 to 13.75 min) from finishing the CZT scan until the consecutive scan on the NaI camera was initiated. Vice versa, for “NaI scan first, CZT scan second”, the median delay was 9 min (IQR, 8 to 9 min). All acquisitions were performed within the time frame of 3 to 6 h post-injection as recommended by the EANM guideline [12]. To further address photon decay, count numbers were corrected for decay during the delay between the two SPECT acquisitions.

### Visual assessment

First, all images were pseudonymized and then randomized. These images were visually rated separately by two experienced readers (> 5 years of experience in dopamine transporter SPECT imaging). Apart from the images themselves, readers were blinded for any further information, e.g. concerning the patients’ clinical characteristics, camera system or semiquantitative data. Subjects were binarized by the readers into two subgroups “non-neurodegenerative PS” and “neurodegenerative PS”. In case of disagreement between two readers, the images in question were re-evaluated in a common reading session to reach consensus.

### Semiquantification

Specific binding ratios (SBR) were computed by DaTQUANT’s automated quantification process. The subject’s SPECT image is registered to a template located in Montreal Neurological Institute (MNI) space. The template is derived from the SPECT and magnetic resonance images of healthy controls from the ENC-DAT project [20]. After the registration process volumes of interest (VOI) for striatum, putamen, caudate and an occipital background region are superimposed on the SPECT scan. The VOIs are then used for SBR calculation according to the following formula: SBR of region = (mean count density in region-of-interest – mean count density in background region)/mean count density in background region. In addition to SBR analysis, z-scores were calculated by using mean and standard deviation of the SBR from the respective normal database. The following formula was applied: *z*-score = (individual SBR − mean SBR in the respective normal database)/standard deviation of SBR in the respective normal database. Individuals with a z-score lower than -2 were defined as “subjects with neurodegenerative PS”.

### Crossover comparison CZT vs. Nal

For the crossover comparison of CZT vs. NaI, count rates, SBR, and z-scores of the 32 subjects imaged on both cameras were analyzed. Only the standard NaI-derived NDB (“GE NDB”) was used for the crossover group’s z-score calculation.

### Dedicated CZT NDB

From the non-neurodegenerative PS subgroup, 25 individuals were randomly selected and defined as CZT NDB group. Clinical follow-up > 24 months after [^123^I]FP-CIT SPECT of these subjects was used as confirmation of non-neurodegenerative PS. Thus, a new, dedicated CZT normal database was created in DaTQUANT with SPECT data of these 25 individuals (CZT NDB). The approach of using a NDB derived from subjects with non-neurodegenerative PS is reasonable since false-negative cases can mostly be attributed to subjects without evidence of dopaminergic deficit (SWEDD) [21, 22].

### Test group

The remaining 48 of 73 subjects (14 visually rated as non-neurodegenerative PS and 34 as neurodegenerative PS) were labeled as “test group”. In case of discordance between visual rating and semiquantification, the visual assessment was confirmed by clinical follow-up for the respective individuals. For the subjects in the test group, z-score calculation was performed with both the GE NDB and the new, dedicated CZT NDB. DaTQUANT does not allow disabling age correction when using its manufacturer-provided normal database (GE NDB), so only age-corrected values were considered for further comparison between CZT and GE NDB to ensure comparability.

**Fig. 1:**
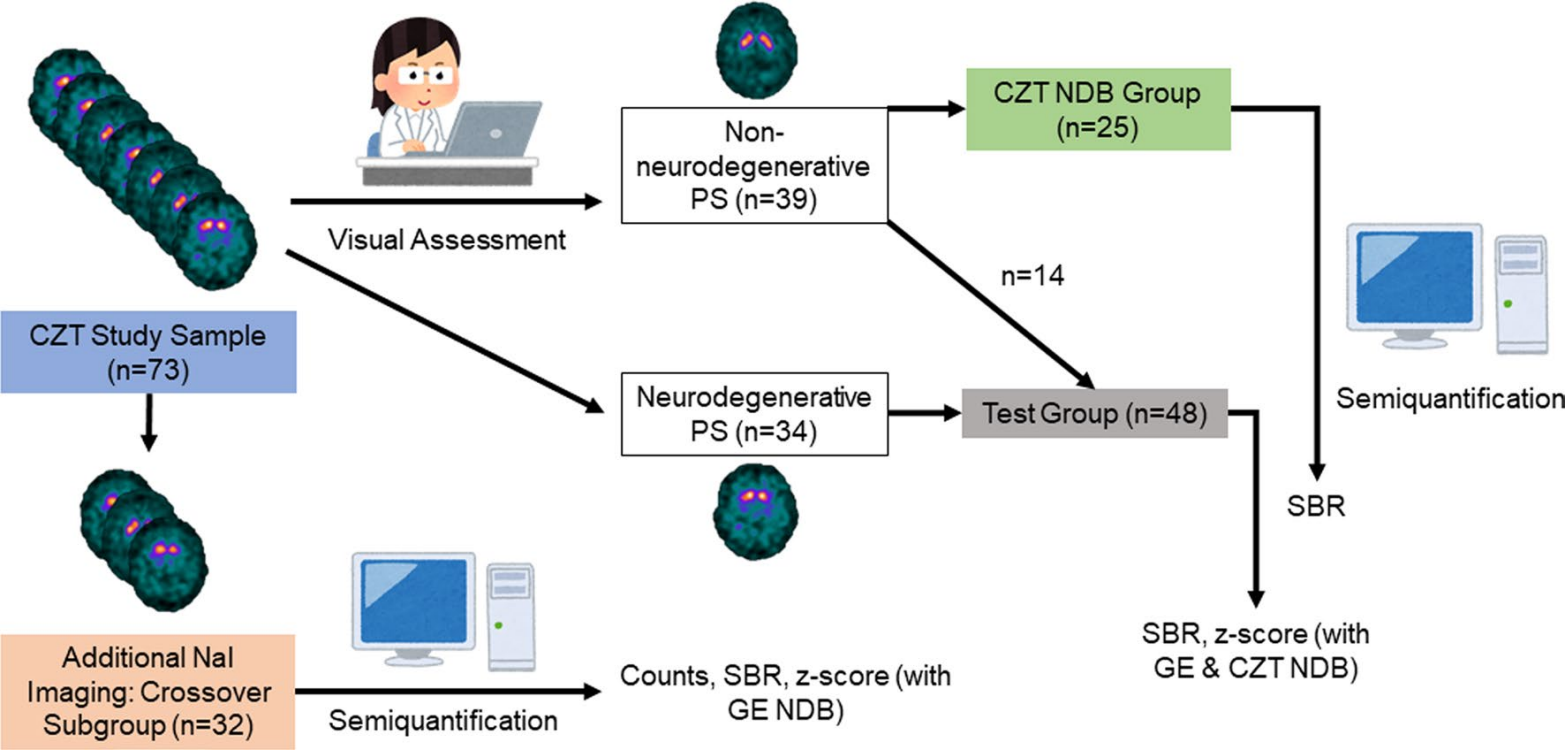
Flowchart of study design and composition of subgroups

Figure 1 summarizes the design of the study and the composition of the different subgroups.

### Statistical analysis

All statistical analysis was performed with SPSS (IBM SPSS Statistics v.25). Based on Shapiro–Wilk test, non-normal distribution was assumed, and for descriptive analysis median, IQR and range were used.

For all subjects, SBR analysis was limited to the putamen with the lowest SBR. This approach was chosen since reduction in the contralateral putamen showed the largest effect size on the loss of dopamine function in neurodegenerative PS patients in comparison to the ipsilateral putamen or bilateral caudate [19].

In the crossover subgroup, median differences in counts, SBR and z-score between the CZT and NaI camera were analyzed by Wilcoxon signed-rank test.

For subjects in the test group, absolute differences between both normal databases for z-scores were examined using a Wilcoxon signed-rank test. Discordances in z-scores between both normal databases were analyzed with McNemar’s test. The *p* values were calculated with the mid-*P* version of McNemar’s test using R (v4.2.1) as recommended by Pembury Smith and Ruxton based on its advantageous performance regarding power and type I error rate [23]. Discordances between visual rating and z-scores of each database were also assessed with McNemar’s test. The performance of putaminal z-score using the CZT NDB to identify individuals with neurodegenerative PS was examined by receiver operating characteristic (ROC) analysis. Optimal cut-off for differentiation between non-neurodegenerative and neurodegenerative PS was determined from ROC analyses by maximization of Youden’s index J = sensitivity + specificity – 1.

## Results

### Study sample

Age and gender proportions of the respective subgroups are summarized in Table 1.

**Table 1:** Age and gender balance of the individuals in all subgroups. Age difference between the NDB and test group was not significant (Mann–Whitney *U* test, *p* = 0.93)

	Count	Median age (IQR)	Proportion female
*Total sample*	73	67 (61 to 75)	0.37 (27/73)
Non-neurodegenerative PS	39	71 (60 to 75)	0.41 (16/39)
Neurodegenerative PS	34	67 (62 to 75)	0.32 (11/34)
*Crossover group*	32	67 (57 to 77)	0.38 (12/32)
Non-neurodegenerative PS	16	68 (59 to 78)	0.50 (8/16)
Neurodegenerative PS	16	66 (54 to 77)	0.25 (4/16)
*CZT NDB group*	25	67 (64 to 75)	0.52 (13/25)
*Test group*	48	67 (60 to 76)	0.29 (14/48)
Non-neurodegenerative PS	14	73 (56 to 77)	0.21 (3/14)
Neurodegenerative PS	34	67 (62 to 75)	0.32 (11/34)

### Crossover comparison CZT vs. Nal

The crossover group of 32 subjects who underwent additional imaging on the NaI-based system was by chance evenly split into 16 individuals visually assessed as non-neurodegenerative PS and 16 individuals visually assessed as neurodegenerative PS. In the visual rating of the 64 images (one CZT and one NaI image per subject), the two independent readers disagreed on 4 images (6%). These images belonged to the same two patients with their respective CZT and NaI scan. Thus, discrepant consensus was caused by borderline findings in these cases and not due to a different visual impression between the two cameras. The images in question were re-evaluated in a joint reading session to reach consensus.

Median total counts with the CZT camera were 2,093,800 (IQR, 1,883,091 to 2,507,879; range, 1,392,551–3,422,141) vs. 1,543,868 with the NaI system (IQR, 1,370,914–1,783,839; range, 1,104,400–2,967,721), resulting in a median difference of 561,812 counts (IQR, 424,897–697,739; range, −102,752–1,213,658; Wilcoxon signed-rank test, *p* < 0.001). However, median counts in the background VOI were similar for both cameras with 69,272 (IQR, 57,875 to 82,101; range, 48,754–129,215) for CZT vs. 71,190 (IQR, 57,582–87,412; range, 35,060–142,547) for NaI. The median difference of CZT vs. NaI was -570 counts which was not statistically significant (IQR, −9,870–8,680, range; −21,136–14,847; Wilcoxon test signed-rank, *p* = 0.61).

**Fig. 2:**
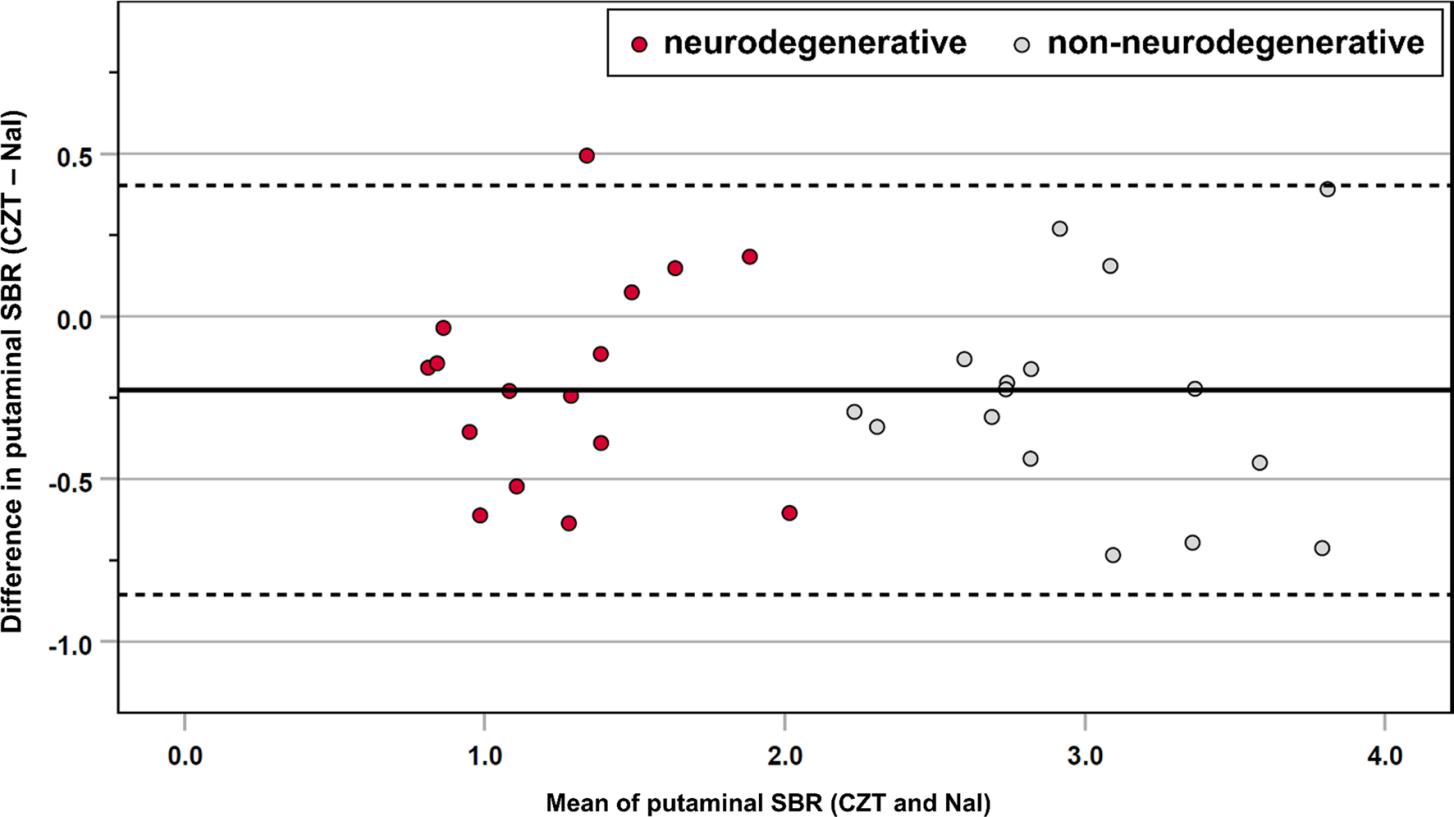
Bland–Altman plot for putaminal SBR between the CZT and NaI camera system in the crossover group. Subjects visually rated as neurodegenerative PS are indicated by red dots, subjects visually rated as non-neurodegenerative PS by gray dots

A median of 29,571 (IQR, 18,387–36,572; range, 11,993–71,578) counts was registered in the putaminal VOI by the CZT system, compared to 31,739 (IQR, 21,205–39,053; range, 14,106–74,113) counts by the NaI system. This resulted in a median difference of 2,324 less counts in the CZT system compared to NaI (IQR, –5,454–961; range, –8,312–4,531; *p* = 0.002). Regarding putaminal SBR, CZT measured a lower median value of 2.03 (IQR, 1.02–2.74; range, 0.99–4.12) than NaI with 2.35 (IQR, 1.38–2.98; range, 0.88–4.14), resulting in a median difference of −0.23 (IQR, −0.45 to −0.06; range, −0.73–0.49; *p* = 0.001). SBR differences between the two camera systems are illustrated in Fig. 2.

A median putaminal z-score of −0.61 (IQR, −3.46–1.33; range, −4.78–3.40) using the GE NDB was calculated for the crossover group’s data of the CZT system, −0.51 (IQR, −2.69–1.69; range, −4.39–4.44) for the NaI system. The resulting median difference of the CZT vs. NaI camera systems was −0.46 (IQR, −0.94 to –0.04; range, −1.68–1.43; *p* = 0.011).

When correlating these z-scores with the visual assessment for neurodegenerative PS (putaminal z-score < −2), there were 7 discordant findings in 6 subjects who were imaged on both systems. For the CZT system, 3 discordant cases could be identified (McNemar’s test, *p* = 0.57), and 4 discordant cases for the NaI system (*p* = 0.71). For one subject, the z-scores of both systems showed discordance with the visual rating, the other discordant cases were either exclusive to the NaI or CZT system. This discrepancy between both camera systems was not significant (*p* = 1.0). For reference, when comparing the visual rating of CZT scans with the CZT z-scores using its dedicated CZT NDB instead of the GE NDB, there were no discordant findings among the 32 subjects of the crossover group.

### CZT NDB group vs. test group for CZT scans: SBR analysis

In the CZT NDB group, median putaminal SBR was 2.9 (IQR, 2.6 to 2.9; range, 2.3–4.0). For the 14 individuals with non-neurodegenerative PS in the test group, it was 2.72 (IQR, 2.3–3.1; range, 2.1–3.3) whereas the 34 subjects with neurodegenerative PS had a median putaminal SBR of 1.25 (IQR, 0.9–1.6; range, 0.7–2.9).

### Test group for CZT scans: z-score analysis

When calculating the z-score for CZT scans by using the CZT NDB for the non-neurodegenerative PS subjects in the test group, the median z-score of the putamen was −0.79 (IQR, −1.7 to −0.4; range, −2.1–0.37). The neurodegenerative PS subjects had a median putaminal z-score of −4.36 (IQR, −5.0 to −3.7; range, −6.4 to −0.1).

**Fig. 3:**
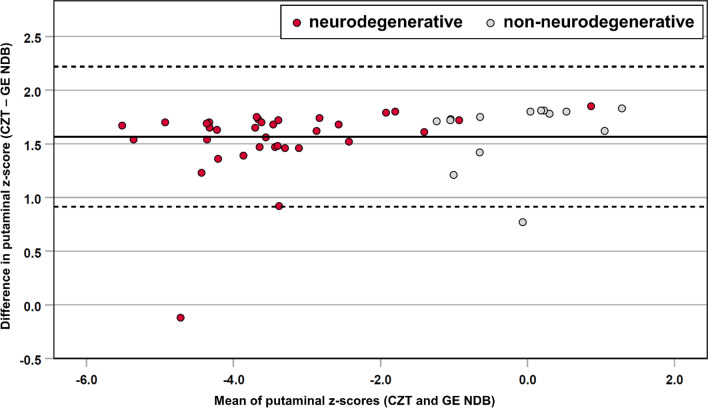
Bland–Altman plot for putaminal z-scores using the CZT NDB and GE NDB, respectively. Subjects visually rated as neurodegenerative PS are indicated by red dots, subjects visually rated as non-neurodegenerative PS by gray dots

Using the GE NDB for CZT scans, the median putaminal z-score was 0.63 (IQR, −0.2–1.3; range, −0.4–2.2) for the individuals with non-neurodegenerative PS and −2.79 (IQR, −3.5 to −2.0; range, −4.8–1.8) for the subjects with neurodegenerative PS. Z-score differences between both databases are illustrated in Fig. 3. The outlier with a mean CZT and GE NDB putaminal z-score of −4.7 represents a subject with advanced neurodegenerative PS at the young age of 35 years, confirmed by clinical follow-up. The misalignment with the other cases can be attributed to the age characteristic of the CZT NDB cohort and the consequent failure of age correction. The second case worth highlighting is a subject whose mean putaminal z-score was 0.9, but who was visually assessed as “neurodegenerative PS” due to a distinct reduction of DAT density in the left putamen (rightmost red dot in Fig. 3). Diagnosis of neurodegenerative PS was confirmed by clinical follow-up.

Calculating the difference between the putaminal z-scores of both normal databases resulted in the median of 1.68 (IQR, 1.5−1.7; range, −0.1−1.9; Wilcoxon signed-rank test, *p* < 0.001). Table 2 summarizes the z-scores using the CZT NDB and GE NDB for CZT scans and the resulting absolute differences for the putamen.

**Table 2:** Z-scores of the CZT NDB and GE NDB for CZT scans and their absolute difference for the putamen (*n* = 48)

z-score	Median	Range	IQR
CZT NDB	−3.84	−6.4 to 0.4	−4.6 to −1.7
GE NDB	−2.46	−4.8 to 2.2	−3.1 to −0.1
Absolute difference	1.68 (*p* < 0.001)	−0.1 to 1.9	1.5 to 1.7

### Visual rating vs. semiquantification: Discordant cases

Separating subjects of the test group with neurodegenerative PS from individuals with non-neurodegenerative PS by means of visual rating and semiquantification (putaminal z-score < -2) resulted in 11 discordant findings. These discordant findings occurred in 9 of 48 subjects for both CZT NDB and GE NDB in their CZT-derived data (Tables 3 & 4). When using CZT NDB, there was a total of 3 discordant cases among 48 subjects compared to the visual rating (6%, McNemar’s test, *p* = 0.63). For the GE NDB, the total was 8 of 48 (17%, *p* = 0.004). One of these discordant cases was exclusive to the CZT NDB, 6 to the GE NDB, and 2 occurred for both NDB. This discrepancy between both NDB was not significant (*p* = 0.07).

**Table 3:** Crosstab comparing the visual rating in the test group to putaminal z-scores with the CZT NDB and GE NDB

Visual rating	CZT NDB		GE NDB	
	z-score ≥ -2	z-score < -2	z-score ≥ -2	z-score < -2
Normal	13 (27%)	1 (2%)	14 (29%)	0
Abnormal	2 (4%)	32 (67%)	8 (17%)	26 (54%)

**Table 4:** Comparison of CZT NDB and GE NDB z-scores along with visual rating for the putamen in the test group (*n* = 48); *p* values were calculated by using McNemar’s test

	CZT NDB vs. visual	GE NDB vs. visual	CZT NDB vs. GE NDB
Discordant cases	3 (*p* = 0.63)	8 (*p* = 0.004)	1 vs. 6 (*p* = 0.07)

**Fig. 4:**
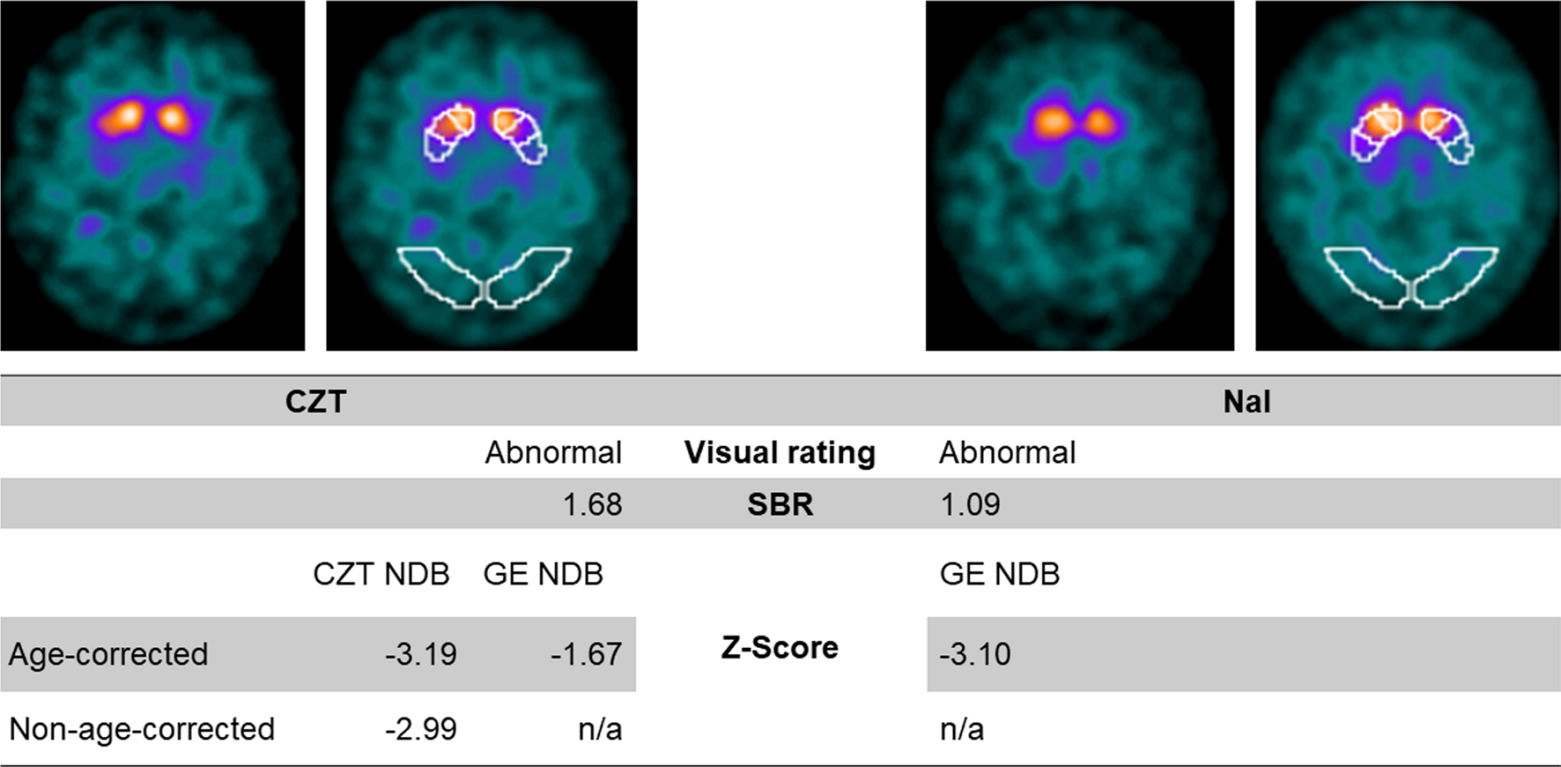
FP-CIT SPECT images of a 65-year-old male first acquired on a NaI (right) and directly afterward on a CZT (left) system. The subject was visually rated as a neurodegenerative PS. For the CZT data, z-scores calculated with the CZT NDB corresponded with the visual assessment, whereas the utilization of the GE NDB resulted in discordance. Semiquantitative results of the NaI system were in line with the visual rating

Furthermore, it is worth noting that 3 of the 9 subjects with discordant findings were part of the crossover group. The discordant findings of these 3 individuals only occurred when using the GE NDB on their CZT scans. However, when applying the GE NDB on their NaI-based data, two of them had z-scores of -2.04 and -3.1, respectively, which was in line with the visual rating of neurodegenerative PS. The other subject’s z-score was -1.72 and remained in contrast to their visual rating as neurodegenerative PS, even for their NaI scan with the GE NDB. In all 9 individuals with discordant findings, clinical follow-up > 24 months after [^123^I]FP-CIT SPECT confirmed the visual assessment. Figure 4 illustrates one case with discordant findings.

### ROC analysis

**Fig. 5:**
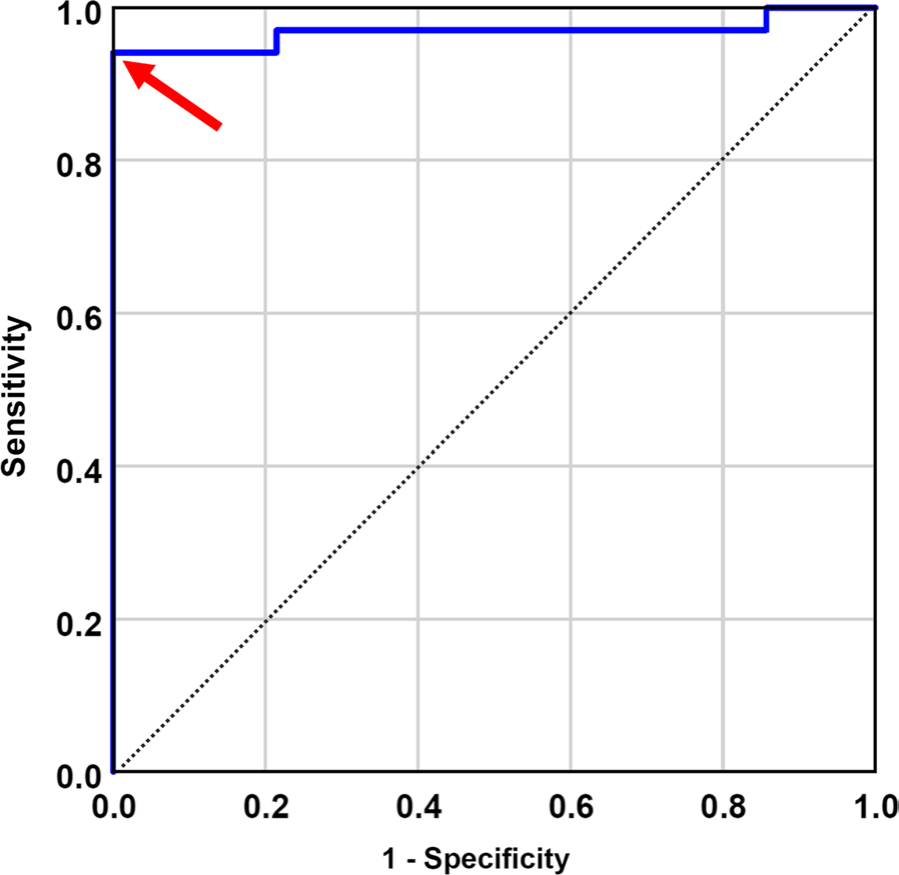
Blue ROC curve for the test group’s putaminal z-scores of the CZT NDB to classify neurodegenerative vs. non-neurodegenerative PS (reference: visual rating). The dotted line serves as reference line. The optimal putaminal z-score cut-off for the CZT NDB (− 2.15; Youden’s index J = 0.94) is marked with a red arrow

ROC analysis showed a high performance in the test group for the CZT NDB’s putaminal z-scores for the identification of subjects with neurodegenerative PS (Fig. 5). Area under the ROC curve for the CZT NDB was 0.968 (standard error, 0.26; 95% CI, 0.917 – 1.0). Optimal putaminal z-score cut-off for the CZT NDB was -2.15 with a sensitivity of 94.1% (95% CI 80.3% – 99.3%) and specificity of 100% (95% CI 76.8% – 100%).

## Discussion

To the best of our knowledge, this is the first study to investigate the difference in semiquantification of CZT [^123^I]FP-CIT SPECT data with a commercial NaI-based GE normal database versus a prospectively created CZT normal database. Our primary finding was that the CZT NDB enables superior performance compared to the GE NDB for CZT brain SPECT scans. This resulted in a lower number of discordant cases in correlation to the visual rating (3 vs. 8 out of 48 subjects). Consequently, discordance between CZT-derived putaminal z-scores and visual rating was not significant (*p* = 1.0), while the use of the GE NaI-derived NDB led to significant inconsistencies (*p* = 0.008).

In theory, the comparison of two ideal databases NDB1 and NDB2, represented by mean SBR (M1; M2) and standard deviation (SD1; SD2), can be expressed as difference of the respective z-scores, i.e.

$$z2 - z1 = \frac{{\left( {{\text{SBR}} - M2} \right)}}{{{\text{SD}}2}} - \frac{{\left( {{\text{SBR}} - M1} \right)}}{{{\text{SD}}1}}$$

which translates to the following expression for a given value of SBR:

$$z2 = \frac{SD1}{{SD2}} \cdot z1 + \frac{{\left( {M1 - M2} \right)}}{SD2}$$

Therefore, both z-scores ​​are directly proportional to each other. Comparing two different cameras, mean SBR values are clearly different (M1 ≠ M2) since they are highly dependent on various biological and camera-related factors. However, the variability is in a first approximation comparable in both databases (SD1≈SD2). It represents between-subject variability and, consequently, is more or less independent of the camera type. This means the two databases only differ by a constant:

$$z2 \approx z1 + \frac{{\left( {M1 - M2} \right)}}{SD2}$$

DaTQUANT does not provide the exact characteristics (mean and standard deviation) necessary for the calculation of this constant. Nevertheless, the consistent median difference of 1.68 (IQR, 1.5 to 1.7; *p* < 0.001) between our two NDB infers a measured approximation of the constant. Our findings suggest that the establishment of a CZT-derived NDB achieves semiquantitative values more in line with the visual rating of [^123^I]FP-CIT SPECT images acquired on a CZT-based camera system.

Most likely, the higher consistency of these cameras with a CZT NDB is based on the difference in SBR values compared to a conventional NaI-based system. Using a crossover comparison of both camera systems, the CZT-based system registered more total counts compared to the NaI system, due to the higher sensitivity of the WEHR collimator of the CZT camera (85 cps/MBq for ^99m^Tc) compared to the low-energy high-resolution (LEHR) collimator of the NaI system (74 cps/MBq for ^99m^Tc). Counts in the background VOI, however, were similar for both systems. Surprisingly, a significantly lower median count rate in the putamen of 2,375 (CZT 29,571 vs. NaI 31,739, *p* = 0.002) was read out by DaTQUANT for the CZT camera. Consequently, putaminal SBR of the CZT camera (median, 2.03) was lower by a median of -0.23 than the SBR of the NaI camera (2.35; *p* = 0.001).

This effect can be attributed to the different characteristics of the WEHR collimator compared to the LEHR collimator. According to the manufacturer’s datasheet, the WEHR collimator of the CZT system has a higher septal penetration than the LEHR collimator of the NaI camera (0.55% vs. 0.3% for ^99m^Tc). ^123^I partially emits high-energy photons, which are usually filtered by the LEHR collimator. Having a higher septal penetration in the CZT system, these photons might cause scatter effects. This can result in an elevated background signal compared to subcortical structures and, therefore, to a reduced signal-to-noise ratio. Narrowing the energy window’s width or usage of triple energy window scatter correction might be possible solutions to mitigate these effects. Another option would be the usage of a medium-energy high-resolution sensitivity (MEHRS) collimator. As shown in a study by Ito et al. [24], this collimator demonstrated an improved scatter-signal removal and superior high-energy resolution on ^123^I imaging with a CZT camera in comparison to a low-medium-energy general purpose (LMEGP) collimator of a NaI system.

In turn, the SBR difference between the two detector systems leads to discrepancies in the subsequent semiquantification steps. When comparing the z-scores of the two databases, the usage of the CZT NDB produced consistently lower z-scores with a median absolute difference of 1.68 to the GE NDB.

Crossover comparison between the CZT and NaI system with our small sample size of 32 subjects identified discordance in 6 subjects when using the GE NDB in correlation to the visual assessment of neurodegenerative PS. All discordant cases had a false-negative outcome (abnormal scan vs. putaminal z-score ≥ -2), whereas the [^123^I]FP-CIT SPECT data of the CZT system in combination with its dedicated CZT NDB showed a complete alignment with the visual rating. However, it is worth noting that the putaminal z-score from the CZT NDB produced one false positive result compared to the visual reading in an 80-year-old male. The two readers categorized his scan as normal, and his symptoms were confirmed as non-neurodegenerative by clinical follow-up. Yet, his CZT-based putaminal z-score was -2.09. In comparison, the subject’s z-score calculated when using the GE NDB was -0.38. The case underlines our initial statement that semiquantification should only be considered as a complementary tool to the visual interpretation of the [^123^I]FP-CIT scan, especially in patients with borderline z-scores close to the pathological threshold.

To improve performance of semiquantification when working with the GE NDB on CZT-derived [^123^I]FP-CIT SPECT data, increasing the cut-off for the putaminal z-score to −0.5 can be considered. This number derives from the consistently higher z-scores calculated with the GE NDB (median difference of 1.68 between both NDB). Based on these findings, an increase in the cut-off could prove as a viable option when establishing a dedicated CZT NDB does not seem feasible.

The present study had the following limitations: First, the size of our CZT NDB with 25 individuals was rather limited. However, according to a study by Schmitz-Steinkrüger et al. [22], normal databases for [^123^I]FP-CIT SPECT should be comprised of at least 25 to 30 subjects to warrant a reliable performance of semiquantification. When increasing the size beyond 40, diminishing returns in accuracy were reported. Hence, the lack of statistical significance in regard to discordance between the CZT NDB and GE NDB use is more likely caused by the limited size of our test group with 48 subjects. Second, to evaluate the effects of age correction on semiquantification of our data, we also intended to compare the z-scores between the CZT and GE NDB. However, this was not possible since DaTQUANT does not provide the option to turn off age correction during semiquantification. Nevertheless, we assume that the intra-subject factor of the used NDB does not interact with the between-subject factor age or the z-score. Finally, our normal database was established by using data of subjects who underwent [^123^I]FP-CIT SPECT in cases of clinically uncertain PS. External physicians referred these patients to our center, and their clinical diagnosis after further follow-up served as standard of truth in this study. The referring physicians were not blinded for the results of the [^123^I]FP-CIT SPECT which might have led to bias in their diagnosis and, consequentially, an overestimation in the performance of putaminal SBR.

## Conclusion

Our findings suggest that a camera-specific CZT-derived normal database improves concordance between semiquantification of [^123^I]FP-CIT CZT SPECT data with visual assessment of the DAT status compared to a NaI-based databases used in CZT scanners. The CZT-specific z-scores account for down-scatter effects by the high-energy photons of ^123^I with the different CZT collimators. Therefore, usage of a dedicated CZT normal database should be preferred when performing [^123^I]FP-CIT SPECT on cameras equipped with general purpose CZT detectors.

## Data Availability

The datasets used and analyzed during the current study are available from the corresponding author on reasonable request.

## Abbreviations

CZT: Cadmium zinc telluride
DAT: Dopamine transporter
[^123^I]FP-CIT: N-ω-fluoropropyl-2β-carbomethoxy-3β-(4-I-123-iodophenyl)nortropane
IQR: Interquartile range
NDB: Normal database
PS: Parkinsonian syndrome
SBR: Specific binding ratio
SPECT: Single-photon emission computed tomography
VOI: Volume of interest
